# PRMT3 in cancer: arginine methylation as a driver of tumor metabolism, immune evasion, and therapeutic resistance

**DOI:** 10.3389/fimmu.2026.1828765

**Published:** 2026-05-01

**Authors:** Hui Jin, Hua Huang, Xin Pan

**Affiliations:** 1 Department of Respiratory Medicine, Affiliated People’s Hospital of Jiangsu University, Zhenjiang, Jiangsu, China; 2 Department of Oncology, Zhenjiang Fourth People’s Hospital, Zhenjiang, Jiangsu, China; 3 Zhenjiang 359 Hospital, Zhenjiang, Jiangsu, China

**Keywords:** arginine methylation, immune evasion, PRMT3, therapeutic resistance, tumor metabolism

## Abstract

Protein arginine methyltransferase 3 (PRMT3) is a type I arginine methyltransferase that catalyzes asymmetric dimethylation of arginine residues on diverse substrate proteins. Initially characterized as a regulator of ribosomal protein methylation, PRMT3 has more recently been implicated in several cancer-related processes. Accumulating evidence suggests that PRMT3 is dysregulated in a variety of malignancies, including hepatocellular carcinoma, colorectal cancer, glioblastoma, breast cancer, pancreatic cancer, and non-small cell lung cancer. Through arginine methylation of selected regulatory proteins, PRMT3 has been linked to signaling pathways associated with tumor progression, metabolic adaptation, immune modulation, and therapeutic resistance. Mechanistically, available studies indicate that PRMT3 can regulate RNA-associated networks by methylating RNA-binding proteins and epitranscriptomic regulators such as IGF2BP1 and METTL14, thereby influencing mRNA stability and gene expression programs. In addition, PRMT3 has been reported to contribute to tumor metabolic reprogramming by promoting glycolytic activity and modulating amino acid metabolism through factors including HIF1A, PDHK1, and IDO1. These alterations may support tumor growth and, in some contexts, influence the tumor immune microenvironment. PRMT3 has also been associated with immune evasion, for example through effects on PD-L1 expression and innate immune signaling pathways such as cGAS–STING. Moreover, emerging evidence links PRMT3 to therapeutic resistance through mechanisms involving oncogenic transcript stabilization, immunometabolic remodeling, and drug efflux regulation. In this review, we summarize the current understanding of PRMT3 structure, catalytic mechanisms, and biological functions in cancer. We further discuss its emerging roles in metabolic regulation, immune suppression, and therapy resistance, while distinguishing mechanisms directly supported within specific cancer contexts from broader conceptual models inferred across studies. Overall, current evidence supports PRMT3 as an emerging and context-dependent regulator of tumor biology and a potential target for anticancer therapy.

## Highlights

- PRMT3-mediated arginine methylation regulates RNA stability, metabolic reprogramming, and immune signaling in cancer.
- PRMT3 promotes tumor progression and therapy resistance through IGF2BP1, METTL14, HIF1A, PDHK1, and IDO1-dependent pathways.
- Targeting PRMT3 represents a promising strategy for overcoming metabolic adaptation, immune evasion, and treatment resistance in cancer.

## 1 Introduction

Protein arginine methylation is a widespread post-translational modification that plays a crucial role in regulating diverse cellular processes, including gene transcription, RNA processing, signal transduction, and metabolic homeostasis (1–5). This modification is catalyzed by a family of enzymes known as protein arginine methyltransferases (PRMTs), which transfer methyl groups from S-adenosyl-L-methionine (SAM) to the guanidino nitrogen atoms of arginine residues in target proteins (6–10). Based on the type of methylarginine products generated, PRMTs are generally classified into three groups: type I PRMTs, which catalyze the formation of asymmetric dimethylarginine (ADMA); type II PRMTs, which generate symmetric dimethylarginine (SDMA); and type III PRMTs, which produce monomethylarginine (MMA) (11–14). Through these enzymatic activities, PRMTs regulate a broad spectrum of biological functions, including chromatin remodeling, RNA metabolism, DNA damage response, and cell signaling. Dysregulation of PRMT-mediated arginine methylation has increasingly been recognized as a hallmark of multiple diseases, particularly cancer.

Among the PRMT family members, protein arginine methyltransferase 3 (PRMT3) has emerged as an important regulator of cellular homeostasis and tumor biology (15, 16). PRMT3 belongs to the type I PRMT subfamily and primarily catalyzes the formation of asymmetric dimethylarginine on its substrates. Structurally, PRMT3 contains a conserved catalytic domain and a zinc finger motif that facilitates substrate recognition and protein–protein interactions. Early studies mainly linked PRMT3 to ribosomal protein methylation and ribosome biogenesis, suggesting a role in translational regulation. However, accumulating evidence over the past decade indicates that PRMT3 has broader functions extending far beyond ribosome biology. Increasing numbers of PRMT3 substrates have been identified, including RNA-binding proteins, metabolic regulators, and signaling molecules, highlighting its diverse regulatory capacity in multiple cellular pathways.

Recent studies have revealed that PRMT3 is frequently dysregulated in a variety of human malignancies, including hepatocellular carcinoma, colorectal cancer, glioblastoma, pancreatic cancer, breast cancer, and non-small cell lung cancer. Elevated PRMT3 expression has been associated with enhanced tumor proliferation, metabolic reprogramming, immune evasion, and resistance to therapy (17). Mechanistically, PRMT3 promotes tumor progression by methylating key regulatory proteins that control oncogenic signaling networks. For example, PRMT3-mediated methylation of RNA-binding proteins such as IGF2BP1 stabilizes oncogenic transcripts and contributes to chemoresistance. In addition, PRMT3 has been shown to modulate epitranscriptomic regulators such as METTL14, thereby influencing mRNA methylation and post-transcriptional gene regulation. These findings place PRMT3 at the intersection of arginine methylation and RNA regulatory networks.

Beyond RNA regulation, PRMT3 has also been implicated in the metabolic reprogramming of cancer cells (15, 16). Metabolic alterations are a hallmark of tumor development, enabling malignant cells to sustain rapid proliferation and adapt to the nutrient- and oxygen-limited tumor microenvironment. Emerging evidence suggests that PRMT3 regulates metabolic pathways such as glycolysis and amino acid metabolism by modulating key metabolic regulators, including HIF1A and PDHK1. Through these mechanisms, PRMT3 contributes to the metabolic plasticity of tumor cells and supports tumor growth and survival under stress conditions. Importantly, recent studies have highlighted a critical role of PRMT3 in shaping the tumor immune microenvironment. PRMT3 can influence immune evasion by regulating immune checkpoint molecules and immunometabolic pathways. For instance, PRMT3-driven metabolic changes can promote PD-L1 expression, thereby suppressing cytotoxic T cell activity. Moreover, PRMT3 has been shown to modulate innate immune signaling pathways such as the cGAS–STING axis through the methylation of mitochondrial stress-related proteins. These findings suggest that PRMT3 functions not only as a metabolic regulator but also as an immunomodulatory factor that contributes to tumor immune escape. Another important aspect of PRMT3 biology is its involvement in therapeutic resistance (18). Cancer cells frequently develop resistance to chemotherapy, radiotherapy, and targeted therapies through adaptive molecular mechanisms. Increasing evidence indicates that PRMT3-mediated arginine methylation plays a role in these resistance processes by stabilizing oncogenic factors, reprogramming metabolic pathways, and creating an immunosuppressive microenvironment. These discoveries have raised considerable interest in targeting PRMT3 as a potential anticancer strategy. Indeed, recent efforts have focused on the development of PRMT3 inhibitors and degraders, highlighting the therapeutic potential of targeting PRMT3-mediated signaling networks. Collectively, these findings position PRMT3 as a multifunctional regulator that integrates epigenetic signaling, RNA regulation, metabolism, and immune responses in cancer. Understanding the molecular mechanisms underlying PRMT3-mediated arginine methylation may therefore provide new insights into tumor biology and reveal novel opportunities for therapeutic intervention.

This review summarizes the emerging roles of PRMT3 in cancer, with particular emphasis on its context-dependent links to tumor metabolism, immune evasion, and therapeutic resistance. Unlike previous reviews that have mainly addressed PRMT family biology or the general functions of PRMT3, the present manuscript specifically integrates recent evidence connecting PRMT3 with metabolic adaptation, immune regulation, and treatment response in cancer. We further discuss the molecular basis of these reported functions and highlight both the therapeutic potential and the current challenges of targeting PRMT3 in cancer.

## 2 Structural features and catalytic mechanisms of PRMT3

PRMT3 is a member of the type I PRMT family that catalyzes the transfer of methyl groups to arginine residues on substrate proteins. Through this enzymatic activity, PRMT3 regulates diverse biological processes including protein–protein interactions, RNA metabolism, signal transduction, and cellular stress responses. Understanding the structural organization and catalytic mechanism of PRMT3 is essential for elucidating its biological functions and for developing therapeutic strategies targeting this enzyme.

### 2.1 Structure of PRMT3

PRMT3 shares a conserved structural architecture with other members of the PRMT family but also possesses unique features that determine its substrate specificity and regulatory functions. Structurally, PRMT3 consists of a central catalytic domain, a conserved S-adenosyl-L-methionine (SAM)-binding pocket, and additional structural elements that facilitate dimerization and substrate recognition (19, 20). The catalytic core of PRMT3 contains a Rossmann fold that forms the active site responsible for methyl group transfer. This domain binds the methyl donor SAM and positions the target arginine residue of substrate proteins for methylation. Within the catalytic domain, several conserved motifs—including the double E loop and the THW loop—play critical roles in coordinating the methyl transfer reaction and stabilizing the transition state during catalysis. These structural elements are highly conserved across PRMT family members and are essential for enzymatic activity.

Adjacent to the catalytic core lies the SAM-binding pocket, which accommodates the methyl donor molecule S-adenosyl-L-methionine. The binding of SAM is essential for PRMT3 catalytic function, as it provides the methyl group that is transferred to the guanidino nitrogen atoms of arginine residues (21–23). Structural studies have demonstrated that the SAM-binding site forms a well-defined cavity within the catalytic domain, enabling precise positioning of the cofactor and facilitating efficient methyl group transfer. Following methyl transfer, SAM is converted to S-adenosyl-L-homocysteine (SAH), which subsequently dissociates from the enzyme. Another important structural feature of PRMT3 is its ability to form homodimers. Dimerization is a common characteristic of PRMT enzymes and is required for their catalytic activity. In PRMT3, dimer formation stabilizes the active conformation of the enzyme and creates an extended surface for substrate binding. Structural analyses suggest that residues located at the dimer interface contribute to enzyme stability and proper alignment of catalytic residues. Disruption of dimerization can significantly impair methyltransferase activity, highlighting the importance of this structural arrangement for PRMT3 function. In addition to these conserved catalytic features, PRMT3 also contains structural elements that facilitate substrate recognition and interaction with regulatory proteins. These structural components enable PRMT3 to interact with a variety of cellular proteins, allowing it to participate in diverse signaling pathways. As a result, PRMT3 is capable of modifying substrates involved in translation, RNA processing, metabolism, and immune signaling.

### 2.2 Enzymatic activity

PRMT3 catalyzes the methylation of arginine residues through the transfer of methyl groups from SAM to the guanidino nitrogen atoms of arginine side chains. As a type I PRMT enzyme, PRMT3 primarily generates asymmetric dimethylarginine (ADMA), although monomethylarginine can also be produced as an intermediate during the methylation process. The formation of ADMA alters the physicochemical properties of arginine residues, which can influence protein conformation, protein–protein interactions, and the binding affinity of regulatory complexes. Through this enzymatic activity, PRMT3 exerts broad regulatory effects on cellular signaling networks (24–26). Arginine methylation can modulate protein stability, localization, and interaction with other molecules, thereby influencing downstream biological processes. In cancer cells, PRMT3-mediated arginine methylation has been shown to regulate key proteins involved in tumor growth, metabolic adaptation, and immune evasion.

One of the earliest identified substrates of PRMT3 is ribosomal protein S2 (RPS2), which plays an important role in ribosome assembly and translational control. Methylation of RPS2 by PRMT3 contributes to proper ribosome biogenesis and efficient protein synthesis (27, 28). This early discovery initially linked PRMT3 to translational regulation and ribosomal function. Subsequent studies have revealed that PRMT3 also targets a variety of RNA-binding proteins, expanding its role in post-transcriptional gene regulation. For example, PRMT3-mediated methylation of RNA-binding proteins such as IGF2BP1 can enhance the stability of oncogenic transcripts and promote tumor progression (29–33). By modifying RNA regulatory proteins, PRMT3 influences RNA metabolism, including mRNA stability, translation, and epitranscriptomic regulation. In addition to RNA regulatory proteins, PRMT3 has been shown to modify metabolic regulators and signaling molecules that control cellular metabolism. For instance, PRMT3-mediated methylation of proteins involved in metabolic pathways can promote glycolytic reprogramming and enhance tumor cell adaptation to metabolic stress. These findings highlight an emerging role for PRMT3 in linking arginine methylation to metabolic regulation in cancer.

### 2.3 Substrate recognition preferences and the unique role of the C2H2 zinc finger domain

Like other members of the PRMT family, PRMT3 catalyzes arginine methylation on protein substrates that often contain arginine-rich regions, including RGG/RG- or GAR-like motifs (34, 35). A classical example is ribosomal protein S2 (rpS2), one of the earliest validated physiological substrates of PRMT3, which contains an N-terminal GAR motif. This observation suggests that PRMT3 shares, at least in part, the general PRMT preference for glycine- and arginine-rich substrate environments (36). However, substrate recognition by PRMT3 cannot be explained solely by linear motif preference. A defining structural feature of PRMT3 is its N-terminal C2H2 zinc finger domain, which distinguishes it from other PRMT family members and appears to play a central role in substrate selectivity. Early biochemical studies showed that deletion or disruption of the zinc finger does not abolish the catalytic activity of PRMT3 toward artificial substrates, but markedly impairs its ability to recognize and methylate physiologic substrates in complex cellular extracts. Subsequent work further demonstrated that the zinc finger is necessary and sufficient for interaction with rpS2, supporting the view that this domain functions as a substrate-recognition module rather than a catalytic center. In this sense, PRMT3 substrate specificity is shaped not only by catalytic site compatibility but also by zinc finger-dependent substrate recruitment.

Importantly, emerging substrate-profiling studies indicate that PRMT3 recognition is likely broader than the canonical RGG/RG paradigm. Identified methylation sites are not uniformly confined to classical glycine-arginine-rich regions, suggesting that PRMT3 can also recognize other arginine-containing sequence contexts (19). Moreover, because the PRMT3 zinc finger is capable of interacting with RNA-associated substrates or protein–RNA complexes, substrate selection may depend on a combination of sequence preference, macromolecular context, and zinc finger-mediated recruitment. This may help explain how PRMT3 is able to engage structurally diverse substrates, including RNA-binding proteins, epitranscriptomic regulators, and transcription-related factors. At present, however, a universal PRMT3 substrate-recognition code has not been fully established. Future structural and biochemical studies will be needed to determine how the zinc finger domain, catalytic groove, substrate sequence, and RNA/protein complex architecture cooperate to confer selective recognition of different PRMT3 substrates.

**Figure 1:**
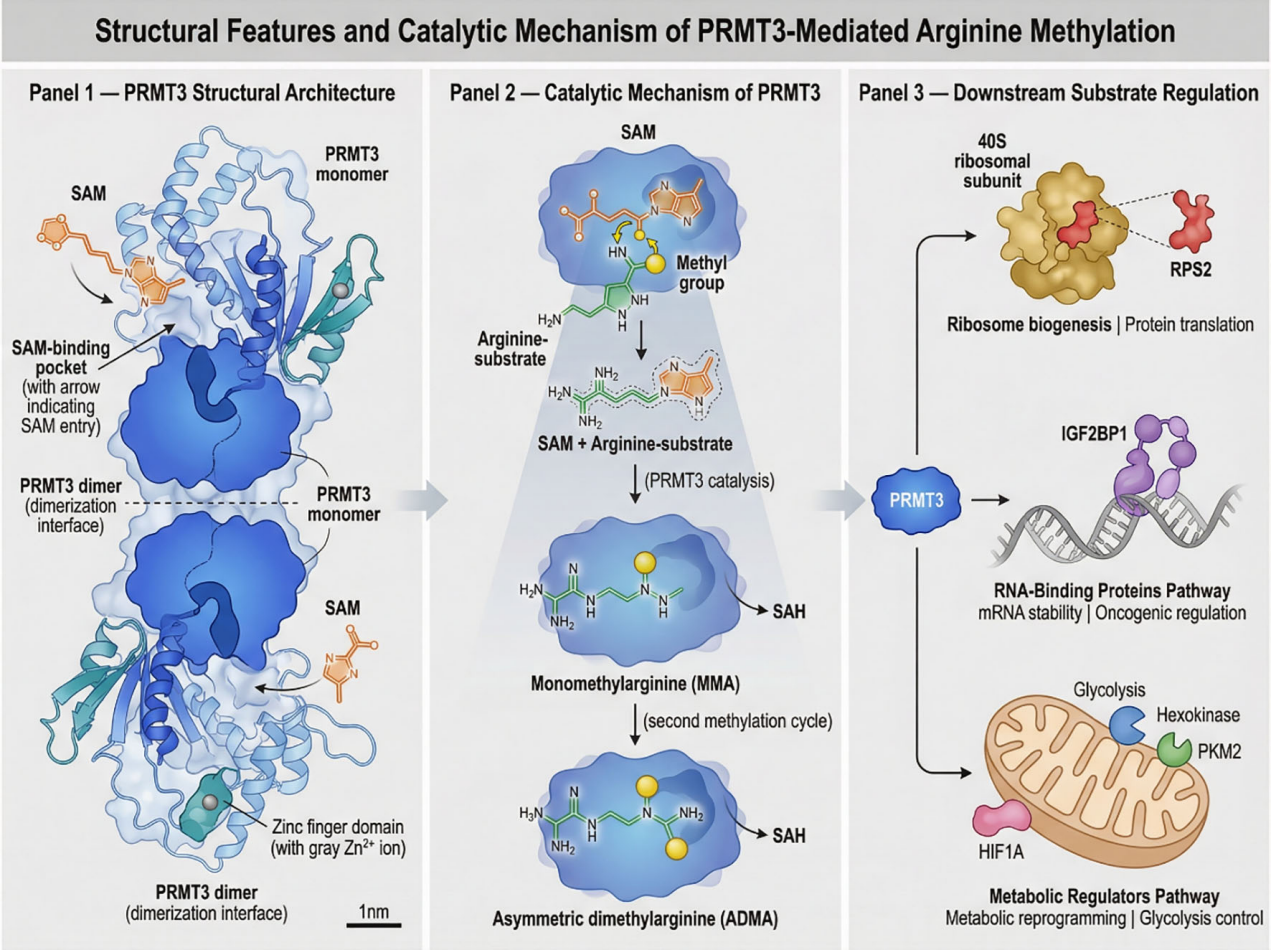
PRMT3 is a type I protein arginine methyltransferase that forms homodimers and catalyzes the transfer of methyl groups from S-adenosyl-L-methionine (SAM) to arginine residues of substrate proteins, generating asymmetric dimethylarginine (ADMA). Through methylation of ribosomal proteins, RNA-binding proteins, and metabolic regulators, PRMT3 modulates translational control, RNA metabolism, and metabolic signaling pathways.

Collectively, PRMT3 enzymatic activity enables the modification of a diverse set of substrates, including ribosomal proteins, RNA-binding proteins, and metabolic regulators. Through these interactions, PRMT3 acts as a multifunctional signaling node that integrates translational control, RNA regulation, and metabolic pathways. The growing list of PRMT3 substrates underscores its importance in coordinating multiple cellular processes and suggests that dysregulation of PRMT3-mediated methylation may contribute to tumorigenesis and cancer progression. **Figure 1** illustrates the structural organization and catalytic mechanism of PRMT3-mediated arginine methylation. It also highlights representative substrate categories through which PRMT3 regulates translation, RNA metabolism, and metabolic signaling.

## 3 PRMT3 expression and clinical significance in cancer

Increasing evidence indicates that PRMT3 is frequently dysregulated in multiple human malignancies and plays a critical role in tumor development and progression. Aberrant expression of PRMT3 has been reported across a wide spectrum of cancer types, including hepatocellular carcinoma, non-small cell lung cancer, colorectal cancer, glioblastoma, pancreatic cancer, and breast cancer. These findings suggest that PRMT3 may function as an important oncogenic regulator and potential biomarker in cancer (37). In addition to its elevated expression in tumors, PRMT3 levels are often associated with poor clinical outcomes, aggressive tumor phenotypes, and resistance to therapy. Understanding the expression patterns and clinical relevance of PRMT3 across cancer types is therefore essential for evaluating its translational potential. The dysregulation and oncogenic functions of PRMT3 across multiple cancer types are summarized in **Table 1**.

**Table 1:** Dysregulation and oncogenic functions of PRMT3 across human cancers.

Cancer type	PRMT3 function	Molecular mechanism	Biological outcome	Reference
Hepatocellular carcinoma (HCC)	Promotes chemoresistance	PRMT3 methylates IGF2BP1 and stabilizes oncogenic mRNAs	Oxaliplatin resistance and tumor progression	Nat Commun, 2023
Hepatocellular carcinoma (HCC)	Immune evasion	PRMT3 activates PDHK1-driven glycolysis and induces PD-L1 expression	Immune suppression	Cell Death Dis, 2025
Non-small cell lung cancer (NSCLC)	Radioresistance	PRMT3 promotes IDO1-driven kynurenine metabolism	Immune suppression and radiation resistance	Cancer Res, 2026
Colorectal cancer	Tumor metabolism	PRMT3 methylates HIF1A and stabilizes c-MYC	Glycolysis and tumor growth	Cell Death Dis, 2021
Glioblastoma	Metabolic adaptation	PRMT3 enhances HIF1A signaling	Glycolysis and tumor progression	Cell Death Dis, 2022
Breast cancer	Tumor aggressiveness	PRMT3 dysregulation in invasive micropapillary carcinoma	Increased tumor invasiveness	Cancer Sci, 2023
Pancreatic cancer	Chemoresistance	PRMT3 methylates hnRNPA1 → ABCG2 upregulation	Drug resistance	Cancers, 2018

### 3.1 PRMT3 dysregulation across cancer types

Recent transcriptomic and proteomic analyses have revealed that PRMT3 expression is significantly altered in multiple cancers. Publicly available cancer datasets, including The Cancer Genome Atlas (TCGA), have demonstrated elevated PRMT3 expression in several tumor types compared with normal tissues, suggesting a potential oncogenic role for this enzyme. Experimental studies further support these observations, showing that PRMT3 overexpression contributes to tumor growth, metabolic reprogramming, and therapy resistance. In hepatocellular carcinoma (HCC), PRMT3 has been reported to promote tumor progression through multiple molecular mechanisms (38). Studies have shown that PRMT3-mediated arginine methylation regulates key oncogenic pathways and contributes to chemotherapy resistance. For example, PRMT3-mediated methylation of IGF2BP1 stabilizes oncogenic transcripts and promotes resistance to oxaliplatin treatment. In addition, PRMT3 has been implicated in metabolic regulation in HCC by activating PDHK1-driven glycolysis, which subsequently increases PD-L1 expression and facilitates immune evasion (39). These findings highlight the multifaceted roles of PRMT3 in promoting tumor growth and immune suppression in liver cancer.

In non-small cell lung cancer (NSCLC), PRMT3 has been shown to drive tumor progression and resistance to radiotherapy. Mechanistically, PRMT3 promotes the activation of indoleamine 2,3-dioxygenase 1 (IDO1), a key enzyme involved in tryptophan metabolism and kynurenine production (40). Activation of the IDO1–kynurenine pathway creates an immunosuppressive tumor microenvironment that inhibits T cell activity and contributes to radioresistance. These findings suggest that PRMT3 may play an important role in regulating immunometabolic pathways in lung cancer. PRMT3 dysregulation has also been documented in colorectal cancer. Several studies have demonstrated that PRMT3 promotes colorectal tumorigenesis by stabilizing key oncogenic transcription factors and metabolic regulators. For instance, PRMT3-mediated methylation of HIF1α enhances its stability and promotes glycolytic metabolism, which supports tumor growth under hypoxic conditions. In addition, PRMT3 has been shown to regulate the stability of the oncogenic transcription factor c-MYC, further contributing to tumor proliferation and progression (41).

In glioblastoma, PRMT3 has been implicated in metabolic reprogramming and tumor aggressiveness. Elevated PRMT3 expression enhances HIF1A signaling and glycolytic activity, enabling glioblastoma cells to adapt to hypoxic tumor microenvironments and sustain rapid proliferation (42). These findings further support the role of PRMT3 in regulating tumor metabolism and promoting malignant progression. Evidence of PRMT3 dysregulation has also been reported in breast cancer and pancreatic cancer. In invasive micropapillary carcinoma of the breast, PRMT3 expression has been associated with increased tumor invasiveness and poor prognosis (43). In pancreatic cancer, PRMT3-mediated methylation of RNA-binding proteins such as hnRNPA1 promotes the expression of the drug transporter ABCG2, leading to enhanced chemoresistance (44). Collectively, these studies demonstrate that PRMT3 overexpression is a common feature across multiple tumor types and contributes to diverse oncogenic processes.

### 3.2 Prognostic value of PRMT3

The clinical relevance of PRMT3 extends beyond its involvement in tumor biology. Increasing evidence suggests that PRMT3 expression levels are closely associated with patient prognosis in several cancers. High PRMT3 expression has been correlated with advanced tumor stage, increased metastatic potential, and shorter overall survival. Clinical studies have shown that elevated PRMT3 expression is frequently associated with more aggressive tumor phenotypes. For instance, PRMT3 overexpression in hepatocellular carcinoma has been linked to increased tumor growth, metabolic activation, and resistance to chemotherapy. Similarly, in colorectal cancer and glioblastoma, high PRMT3 levels correlate with enhanced glycolytic metabolism and tumor progression. These observations indicate that PRMT3 may serve as a marker of tumor aggressiveness and metabolic adaptation. In addition to its association with tumor progression, PRMT3 expression may also reflect the immunological status of the tumor microenvironment. PRMT3-mediated activation of metabolic and immune regulatory pathways, such as PD-L1 expression and IDO1-driven kynurenine metabolism, can create an immunosuppressive microenvironment that supports tumor immune escape (45–49). As a result, tumors with elevated PRMT3 expression may exhibit reduced immune surveillance and poorer clinical outcomes. Together, these findings suggest that PRMT3 expression may serve as an important prognostic indicator across multiple cancer types. Further large-scale clinical studies will be necessary to validate the prognostic value of PRMT3 and determine its utility in clinical practice.

### 3.3 PRMT3 as a potential biomarker

Given its widespread dysregulation and functional importance in cancer biology, PRMT3 has attracted increasing attention as a potential biomarker for cancer diagnosis, prognosis, and therapeutic response prediction (50). Biomarkers that reflect tumor metabolic state, immune status, or treatment resistance can provide valuable information for patient stratification and personalized therapy. PRMT3 expression may serve as a prognostic biomarker to identify patients with aggressive disease or poor outcomes (51). Elevated PRMT3 levels have been associated with enhanced tumor growth, metabolic reprogramming, and immune suppression, all of which contribute to disease progression. Monitoring PRMT3 expression may therefore help identify patients at higher risk of recurrence or treatment failure. In addition, PRMT3 may also function as a predictive biomarker for therapy response. Because PRMT3 regulates pathways involved in chemotherapy resistance, radiotherapy resistance, and immune evasion, tumors with high PRMT3 expression may respond differently to conventional treatments or immunotherapy. For example, PRMT3-driven metabolic and immune regulatory pathways may influence the efficacy of immune checkpoint inhibitors or metabolic-targeted therapies. Furthermore, advances in bioinformatics and machine learning approaches have facilitated the identification of PRMT3-associated biomarker signatures across multiple cancer types. Pan-cancer analyses have suggested that PRMT3 expression may correlate with immune infiltration patterns and tumor metabolic states, further supporting its potential as a biomarker for tumor classification and treatment stratification.

### 3.4 Upstream regulation and context-dependent activation of PRMT3

While increasing evidence has defined the downstream substrate network of PRMT3 in cancer, its upstream regulation remains comparatively underexplored. Current data suggest that PRMT3 is controlled at multiple levels, including post-transcriptional regulation, protein–protein interactions, and context-dependent modulation of enzymatic activity. For example, a recent study in hepatocellular carcinoma showed that the circERBB3/miR-194-5p axis promotes tumor progression by relieving miR-194-5p-mediated repression of PRMT3, thereby increasing PRMT3 expression (38). In addition to expression control, PRMT3 activity is also shaped by its interacting partners. Early biochemical studies demonstrated that binding of ribosomal protein rpS2 enhances PRMT3 methyltransferase activity, whereas interaction with the tumor suppressor DAL-1/4.1B suppresses its enzymatic function (27, 28). Structural studies further indicate that dimerization is required for PRMT3 catalytic activity, suggesting that conformational regulation may be an important determinant of PRMT3 function. Despite these advances, how PRMT3 is activated under tumor microenvironmental stress remains insufficiently defined. Available evidence indicates that metabolic conditions can influence PRMT3 abundance, as iron depletion reduces PRMT3 gene and protein expression, implying that PRMT3 may be sensitive to nutrient-related cues. Moreover, PRMT3 has been positioned functionally downstream of hypoxia- and metabolism-associated programs, including HIF1A stabilization and the PDHK1–lactate–PD-L1 axis, but whether hypoxia, nutrient deprivation, or inflammatory signaling directly induce PRMT3 itself has not yet been systematically resolved (42, 52, 53). Therefore, elucidating the upstream mechanisms that govern PRMT3 expression, activation, and substrate selection under tumor-associated stress conditions represents an important future direction.

**Figure 2:**
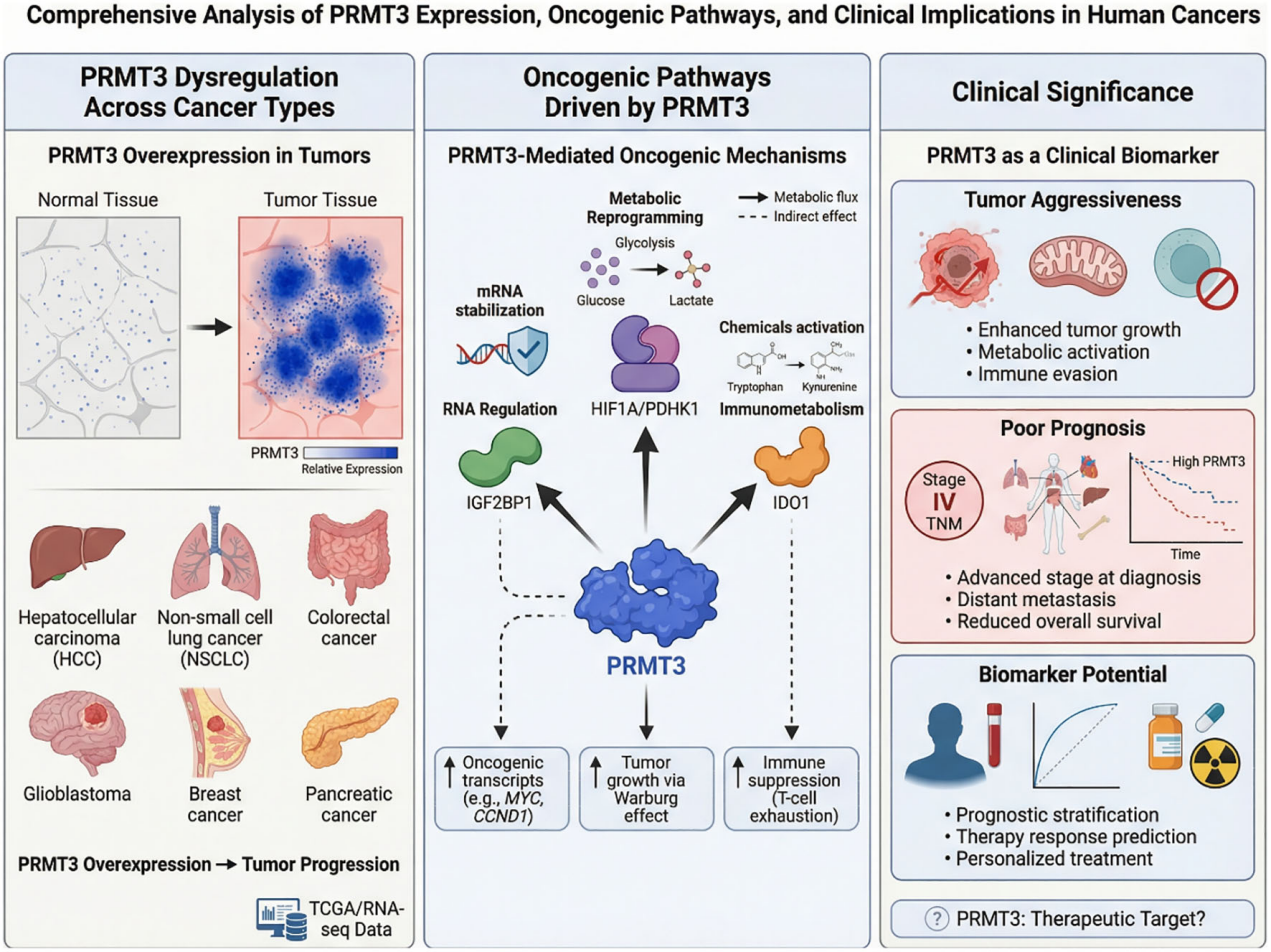
PRMT3 is frequently overexpressed in multiple cancers, including hepatocellular carcinoma, non-small cell lung cancer, colorectal cancer, glioblastoma, breast cancer, and pancreatic cancer. Elevated PRMT3 promotes tumor progression by regulating RNA stability, metabolic reprogramming, and immunometabolic pathways. Clinically, increased PRMT3 expression is associated with aggressive tumor phenotypes, poor prognosis, and therapy resistance, highlighting its potential as a diagnostic, prognostic, and predictive biomarker in cancer.

Taken together, these findings highlight the potential clinical significance of PRMT3 as a diagnostic, prognostic, and predictive biomarker in cancer. Continued research integrating multi-omics datasets and clinical cohorts will be essential to fully elucidate the biomarker potential of PRMT3 and translate these discoveries into clinical applications. **Figure 2** summarizes the dysregulation of PRMT3 across multiple cancer types and its major oncogenic functions. It also highlights the clinical significance of PRMT3 as a biomarker associated with tumor aggressiveness, poor prognosis, and therapy response prediction.

## 4 PRMT3-mediated arginine methylation and RNA regulatory networks

Post-transcriptional regulation of gene expression plays a fundamental role in tumor development and adaptation. In recent years, increasing evidence has revealed that protein arginine methyltransferases participate in the regulation of RNA metabolism by modifying RNA-binding proteins and components of the RNA modification machinery. Among these enzymes, PRMT3 has emerged as an important regulator of RNA regulatory networks in cancer. Through arginine methylation of RNA-binding proteins and epitranscriptomic regulators, PRMT3 can influence mRNA stability, translation efficiency, and RNA modification landscapes. These functions place PRMT3 at a critical intersection between post-translational protein modification and post-transcriptional gene regulation. Recent studies have highlighted two major mechanisms through which PRMT3 regulates RNA-related processes in cancer: methylation of RNA-binding proteins that control mRNA stability, and modulation of epitranscriptomic regulators that shape RNA methylation patterns. These mechanisms collectively contribute to oncogenic gene expression programs and therapeutic resistance. Known substrates of PRMT3 and their downstream signaling pathways in cancer are summarized in **Table 2**.

**Table 2:** PRMT3 substrates and downstream signaling pathways in cancer.

PRMT3 substrate	Type of molecule	Regulatory mechanism	Biological consequence	Cancer type
IGF2BP1	RNA-binding protein	Arginine methylation enhances RNA binding	Stabilization of oncogenic mRNAs	HCC
METTL14	m6A methyltransferase	PRMT3 methylation alters m6A modification	Epitranscriptomic regulation and tumor progression	Endometrial cancer
HIF1A	Transcription factor	PRMT3-mediated stabilization	Activation of glycolytic genes	CRC/GBM
PDHK1	Metabolic enzyme	PRMT3 activates PDHK1 signaling	Glycolytic metabolic shift	HCC
IDO1	Metabolic enzyme	PRMT3 promotes tryptophan metabolism	Immune suppression	NSCLC
hnRNPA1	RNA-binding protein	PRMT3 methylation enhances ABCG2 expression	Drug efflux and chemoresistance	Pancreatic cancer
HSP60	Mitochondrial chaperone	PRMT3 methylation promotes oligomerization	Inhibition of cGAS–STING signaling	Multiple cancers

### 4.1 Methylation of RNA-binding proteins

RNA-binding proteins (RBPs) are essential regulators of post-transcriptional gene expression. By binding to specific RNA sequences or structural motifs, RBPs control multiple aspects of RNA metabolism, including RNA splicing, nuclear export, stability, localization, and translation. Dysregulation of RBPs has been increasingly implicated in cancer progression, as altered RNA stability and translation can amplify oncogenic signaling pathways. Recent evidence indicates that PRMT3 directly regulates the activity of RNA-binding proteins through arginine methylation. One of the most well-characterized examples is insulin-like growth factor 2 mRNA-binding protein 1 (IGF2BP1), a key RNA-binding protein that functions as a reader of N6-methyladenosine (m6A)-modified transcripts. IGF2BP1 recognizes m6A-containing mRNAs and promotes their stability and translation, thereby enhancing the expression of multiple oncogenic transcripts. A recent study demonstrated that PRMT3-mediated arginine methylation of IGF2BP1 plays a critical role in hepatocellular carcinoma progression and chemotherapy resistance (54–58). Specifically, PRMT3 methylates IGF2BP1 at specific arginine residues, enhancing its structural stability and RNA-binding capacity. This modification increases the ability of IGF2BP1 to stabilize oncogenic mRNAs, including transcripts involved in cell proliferation and survival pathways (59–63). As a consequence of enhanced IGF2BP1 activity, oncogenic transcripts are protected from degradation and remain available for translation, leading to increased expression of tumor-promoting proteins. Importantly, this regulatory mechanism has been linked to resistance to oxaliplatin chemotherapy in hepatocellular carcinoma. By stabilizing mRNAs that support tumor cell survival and stress adaptation, PRMT3-mediated IGF2BP1 methylation enables cancer cells to withstand chemotherapeutic pressure (64). This finding highlights an important mechanism by which arginine methylation can regulate RNA-binding protein function and influence cancer cell phenotypes. It also demonstrates how PRMT3 can indirectly control gene expression programs by modifying proteins that regulate RNA stability. Through this mechanism, PRMT3 acts as a post-translational regulator of RNA regulatory networks that promote tumor progression and drug resistance.

### 4.2 PRMT3 and epitranscriptomic regulation

In addition to regulating RNA-binding proteins, PRMT3 also influences the epitranscriptomic machinery that controls RNA modifications. Epitranscriptomics refers to chemical modifications of RNA molecules that regulate RNA metabolism and function without altering the underlying nucleotide sequence. Among these modifications, N6-methyladenosine (m6A) is the most abundant internal modification in eukaryotic mRNA and plays a crucial role in regulating RNA stability, translation, and degradation. The m6A modification is dynamically controlled by three groups of regulatory proteins: methyltransferases (“writers”), demethylases (“erasers”), and m6A-binding proteins (“readers”). METTL14 is a core component of the m6A methyltransferase complex and functions together with METTL3 to catalyze m6A deposition on target transcripts. Through this activity, METTL14 influences multiple cellular processes, including cell differentiation, stress responses, and tumor progression.

Recent research has revealed that PRMT3 can directly regulate the activity of METTL14 through arginine methylation. In endometrial carcinoma, PRMT3-mediated methylation of METTL14 has been shown to alter the function of the m6A methyltransferase complex (65). This modification affects the catalytic activity and stability of METTL14, leading to changes in global m6A modification patterns across the transcriptome. Altered m6A landscapes can profoundly influence gene expression programs by modulating the stability and translation efficiency of numerous transcripts involved in tumor progression. For example, m6A-dependent regulation can affect pathways controlling cell proliferation, apoptosis, and metastasis. Through the methylation of METTL14, PRMT3 can reshape the epitranscriptomic landscape of cancer cells and promote malignant phenotypes (66, 67). An especially intriguing possibility is that PRMT3 may influence both the writing and reading arms of m6A-dependent regulation. As discussed above, PRMT3 has also been shown to modify the m6A reader-associated RNA-binding protein IGF2BP1, thereby affecting RNA stability in cancer-related contexts. This raises the possibility of a PRMT3-centered “writer–reader crosstalk” model, in which PRMT3-mediated regulation of METTL14 could alter m6A deposition on selected transcripts, while PRMT3-dependent effects on IGF2BP1 could influence the recognition and stabilization of those same transcripts. However, to our knowledge, direct evidence supporting this serial connection within the same experimental model is still lacking. Therefore, this potential METTL14–IGF2BP1 axis should currently be regarded as a testable mechanistic hypothesis rather than an established pathway. Future studies should determine whether PRMT3 coordinates transcript-specific m6A deposition and reader-dependent RNA stabilization in a unified regulatory framework.

### 4.3 Integration of arginine methylation and RNA regulatory networks

The discovery that PRMT3 regulates both RNA-binding proteins and m6A methyltransferases highlights a broader role for arginine methylation in controlling RNA regulatory networks. Through the methylation of proteins such as IGF2BP1 and METTL14, PRMT3 can influence both the recognition of modified RNAs and the deposition of RNA modifications themselves (68, 69). This dual regulatory capacity enables PRMT3 to coordinate multiple layers of post-transcriptional gene regulation. On one hand, PRMT3 modifies RNA-binding proteins that directly control mRNA stability and translation. On the other hand, PRMT3 modulates the epitranscriptomic machinery that establishes RNA methylation patterns. Together, these mechanisms create a complex regulatory network that governs RNA fate and gene expression output in cancer cells. From a broader perspective, these findings reveal that PRMT3 functions as an important molecular bridge connecting protein arginine methylation with epitranscriptomic regulation. By integrating protein post-translational modification with RNA regulatory pathways, PRMT3 contributes to the dynamic control of oncogenic gene expression programs.

**Figure 3:**
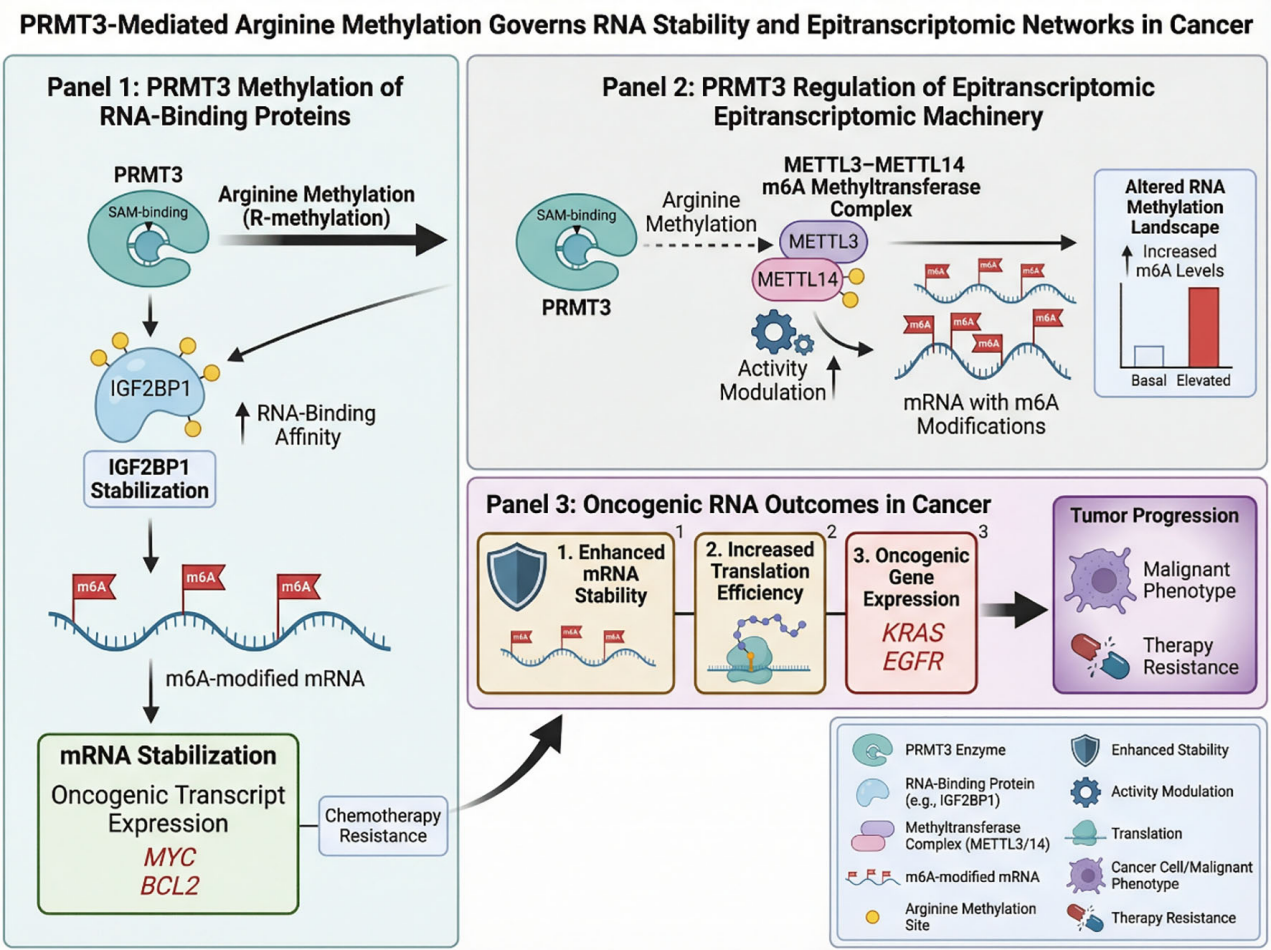
PRMT3 regulates RNA regulatory networks through arginine methylation of RNA-binding proteins and epitranscriptomic regulators. Methylation of IGF2BP1 enhances its RNA-binding ability and stabilizes oncogenic transcripts, while PRMT3-mediated modification of METTL14 influences m6A RNA methylation. These coordinated mechanisms reshape RNA stability and translation, promoting oncogenic gene expression and tumor progression.

Collectively, these studies demonstrate that PRMT3 connects arginine methylation with epitranscriptomic regulation, thereby establishing a regulatory axis that links protein modification, RNA stability, and tumor progression. **Figure 3** illustrates how PRMT3 links arginine methylation with RNA regulatory networks in cancer. By modifying both RNA-binding proteins and m6A methyltransferase machinery, PRMT3 coordinates mRNA stability, epitranscriptomic regulation, and oncogenic gene expression.

## 5 PRMT3 as a driver of metabolic reprogramming

Metabolic reprogramming is a hallmark of cancer and plays a crucial role in supporting tumor growth, survival, and therapeutic resistance. Tumor cells frequently rewire metabolic pathways to adapt to the nutrient-deprived and hypoxic conditions of the tumor microenvironment. These metabolic alterations enable cancer cells to sustain rapid proliferation, maintain redox balance, and evade immune surveillance. Increasing evidence indicates that post-translational modifications, including arginine methylation, contribute significantly to the regulation of metabolic pathways in cancer. Recent studies have identified PRMT3 as an important regulator of metabolic reprogramming. Through arginine methylation of key metabolic regulators and signaling molecules, PRMT3 influences glycolysis, amino acid metabolism, and immunometabolic pathways. These regulatory functions position PRMT3 as a central mediator linking epigenetic signaling to metabolic adaptation in cancer.

### 5.1 Glycolytic reprogramming

One of the most prominent metabolic features of cancer cells is the enhancement of aerobic glycolysis, commonly known as the Warburg effect. In this metabolic state, tumor cells preferentially convert glucose to lactate even in the presence of sufficient oxygen. This metabolic shift allows cancer cells to rapidly generate ATP and metabolic intermediates required for biosynthesis. Glycolytic reprogramming is therefore essential for tumor growth and adaptation to hypoxic conditions. Emerging evidence suggests that PRMT3 contributes to glycolytic activation by regulating key transcription factors and metabolic enzymes involved in glucose metabolism. One important mechanism involves the stabilization of hypoxia-inducible factor 1 alpha (HIF1A), a master transcriptional regulator of the cellular response to hypoxia. HIF1A activates the transcription of numerous genes involved in glycolysis, including glucose transporters and glycolytic enzymes (70).

Recent studies have demonstrated that PRMT3-mediated arginine methylation enhances the stability of HIF1A in cancer cells (53). By methylating regulatory proteins associated with HIF1A signaling, PRMT3 promotes the accumulation of HIF1A and increases the transcription of glycolytic genes. As a result, tumor cells exhibit increased glucose uptake, enhanced glycolytic flux, and elevated lactate production. This metabolic adaptation supports rapid tumor growth and allows cancer cells to survive in hypoxic tumor microenvironments. In addition to regulating HIF1A signaling, PRMT3 has also been shown to modulate glycolytic metabolism through the activation of pyruvate dehydrogenase kinase 1 (PDHK1) (71). PDHK1 is a key metabolic enzyme that inhibits the pyruvate dehydrogenase complex (PDC), thereby preventing the conversion of pyruvate into acetyl-CoA for mitochondrial oxidative phosphorylation (72–75). Activation of PDHK1 therefore promotes the diversion of pyruvate toward lactate production, reinforcing the glycolytic phenotype of cancer cells (76, 77). Recent studies in hepatocellular carcinoma have shown that PRMT3 can activate PDHK1-driven glycolysis, leading to enhanced metabolic plasticity. Increased PDHK1 activity suppresses mitochondrial respiration and shifts cellular metabolism toward glycolysis. This metabolic shift not only supports tumor cell proliferation but also contributes to immune evasion by promoting lactate accumulation in the tumor microenvironment. Elevated lactate levels can inhibit the activity of cytotoxic T cells and natural killer cells, thereby facilitating tumor immune escape.

Together, these findings demonstrate that PRMT3 plays a critical role in regulating glycolytic metabolism through multiple mechanisms, including HIF1A stabilization and PDHK1 activation. By enhancing glycolysis and lactate production, PRMT3 enables cancer cells to adapt to metabolic stress and sustain rapid proliferation.

### 5.2 Amino acid metabolism

In addition to glycolysis, alterations in amino acid metabolism represent another important aspect of tumor metabolic reprogramming. Amino acid metabolic pathways not only provide essential building blocks for protein synthesis but also influence immune regulation within the tumor microenvironment. Among these pathways, tryptophan metabolism has emerged as a key immunometabolic axis in cancer.

Tryptophan catabolism is primarily mediated by indoleamine 2,3-dioxygenase 1 (IDO1), an enzyme that catalyzes the conversion of tryptophan into kynurenine (78–82). Activation of the IDO1–kynurenine pathway has profound immunological consequences. Depletion of tryptophan in the tumor microenvironment suppresses T cell proliferation, while accumulation of kynurenine promotes the differentiation of immunosuppressive regulatory T cells and inhibits cytotoxic immune responses. As a result, activation of this pathway contributes to tumor immune evasion and disease progression. Recent studies have revealed that PRMT3 plays an important role in regulating this immunometabolic pathway. In non-small cell lung cancer, PRMT3 has been shown to promote the expression and activity of IDO1, thereby enhancing kynurenine production. Increased kynurenine levels contribute to the establishment of an immunosuppressive tumor microenvironment that limits the activity of anti-tumor immune cells. Importantly, PRMT3-driven activation of the IDO1–kynurenine pathway has also been linked to resistance to radiotherapy (83–86). Radiation therapy relies in part on the activation of anti-tumor immune responses to eliminate malignant cells. However, the accumulation of kynurenine suppresses immune cell activation and reduces the effectiveness of radiotherapy. By promoting IDO1-mediated tryptophan metabolism, PRMT3 contributes to both immunosuppression and radioresistance in cancer. These findings highlight an emerging role for PRMT3 in the regulation of immunometabolic pathways that integrate metabolism and immune responses. By controlling amino acid metabolism through IDO1 activation, PRMT3 not only supports tumor progression but also reshapes the immune landscape of the tumor microenvironment.

### 5.3 Transition to immunometabolic regulation

Beyond its direct effects on glycolysis and amino acid metabolism, PRMT3 also exerts broader immunometabolic functions in the tumor microenvironment. These integrated effects are discussed in detail in Section 6.2.

**Figure 4:**
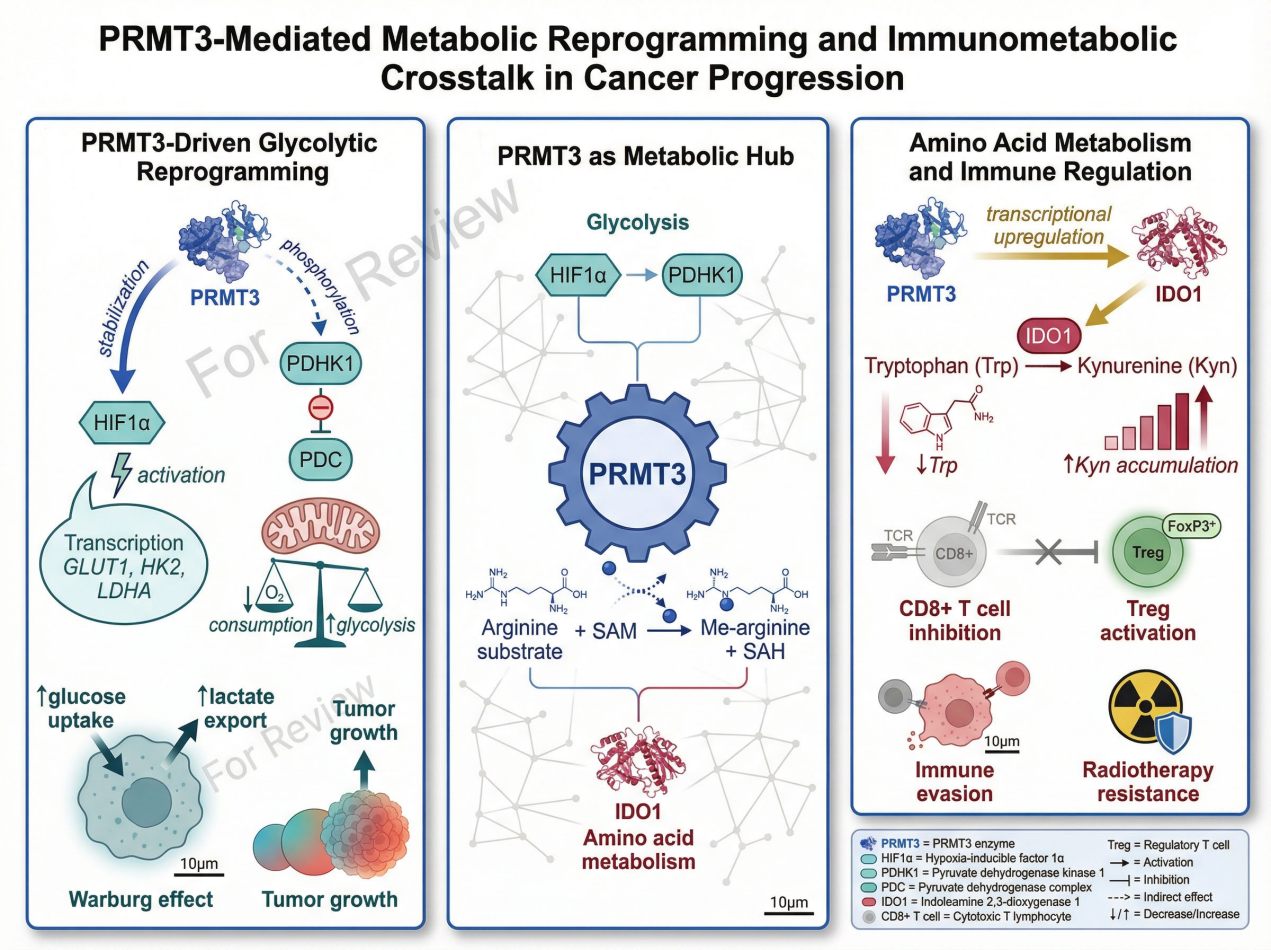
PRMT3 promotes metabolic reprogramming in cancer by regulating glycolysis and amino acid metabolism. PRMT3 enhances HIF1A stabilization and activates PDHK1, leading to increased glycolysis and lactate production. In addition, PRMT3 stimulates IDO1-mediated tryptophan–kynurenine metabolism, creating an immunosuppressive tumor microenvironment. These coordinated metabolic alterations support tumor growth, immune evasion, and therapy resistance.

Collectively, these studies suggest that PRMT3 functions as an important molecular link between metabolic reprogramming and immune regulation. By coordinating glycolysis, amino acid metabolism, and immunosuppressive signaling pathways, PRMT3 contributes to the establishment of a metabolically and immunologically favorable environment for tumor growth. **Figure 4** illustrates how PRMT3 drives metabolic reprogramming in cancer by coordinating glycolytic activation and tryptophan metabolism. Through the regulation of HIF1A, PDHK1, and IDO1, PRMT3 links metabolic adaptation with immune suppression and tumor progression.

## 6 PRMT3 in tumor immune evasion

Tumor immune evasion represents a critical hallmark of cancer and enables malignant cells to escape immune surveillance and sustain long-term tumor growth (87–89). Cancer cells employ multiple strategies to suppress anti-tumor immune responses, including the upregulation of immune checkpoint molecules, metabolic reprogramming that alters the tumor microenvironment, and inhibition of innate immune signaling pathways. Increasing evidence suggests that PRMT3 plays an important role in orchestrating these immunosuppressive mechanisms. Recent studies have revealed that PRMT3 regulates tumor immune escape through multiple pathways. These mechanisms include the metabolic activation of immune checkpoint signaling, suppression of innate immune sensing pathways, and the integration of metabolic and immune regulatory networks within the tumor microenvironment. Through these activities, PRMT3 functions as a molecular regulator that links metabolic reprogramming with immune suppression in cancer.

### 6.1 PD-L1–mediated immune escape

One of the most well-characterized mechanisms of tumor immune evasion involves the activation of immune checkpoint pathways. Programmed death-ligand 1 (PD-L1) is a key immune checkpoint molecule expressed on tumor cells that binds to the PD-1 receptor on T cells, leading to inhibition of T cell activation and cytotoxic function. Upregulation of PD-L1 therefore allows tumor cells to evade immune-mediated destruction and contributes to resistance to anti-tumor immunity (90–94). Recent evidence indicates that PRMT3 can promote PD-L1–mediated immune escape through metabolic reprogramming pathways. The metabolic basis of PRMT3-driven PD-L1 regulation, including the PDHK1–glycolysis–lactate axis, is discussed in Section 6.2.

### 6.2 PRMT3 coordinates immunometabolic reprogramming in the tumor microenvironment

Accumulating evidence suggests that PRMT3 functions not only as a regulator of tumor metabolism or immune evasion in isolation, but also as a key coordinator of immunometabolic reprogramming in the tumor microenvironment (17, 39, 40). Two representative pathways that illustrate this integrative role are PDHK1-driven glycolysis and IDO1-mediated tryptophan–kynurenine metabolism. Rather than acting as separate metabolic events, these pathways converge to create a metabolically and immunologically suppressive niche that promotes tumor progression and therapeutic resistance. One major mechanism involves the PRMT3–PDHK1 axis (39). By activating PDHK1, PRMT3 enhances glycolytic flux and promotes lactate accumulation within the tumor microenvironment. Increased lactate production not only supports metabolic adaptation and tumor growth but also suppresses anti-tumor immunity by impairing CD8+ T-cell function and reinforcing PD-L1-associated immune escape. Thus, PRMT3-driven glycolysis extends beyond a metabolic phenotype and directly contributes to immune suppression. In parallel, PRMT3 also promotes immunometabolic suppression through the IDO1–kynurenine pathway. By enhancing IDO1-mediated tryptophan catabolism, PRMT3 increases kynurenine accumulation, which suppresses effector T-cell activity and favors an immunosuppressive microenvironment (40). This pathway further links amino acid metabolism to tumor immune escape and adaptive treatment tolerance. Importantly, these two pathways converge functionally at the level of therapeutic resistance. Lactate- and kynurenine-enriched tumor microenvironments weaken anti-tumor immune surveillance, reduce the effectiveness of cytotoxic and radiation-induced immune responses, and may limit sensitivity to immunotherapy. Therefore, PRMT3 should be viewed as an immunometabolic bridge that links metabolic alteration to immune dysfunction and, ultimately, to therapy resistance.

### 6.3 Regulation of the cGAS–STING pathway

In addition to regulating immune checkpoint pathways, PRMT3 has also been implicated in the control of innate immune signaling. The cyclic GMP–AMP synthase (cGAS)–stimulator of interferon genes (STING) pathway is a central component of the innate immune response that detects cytosolic DNA and triggers the production of type I interferons and inflammatory cytokines. Activation of the cGAS–STING pathway can promote anti-tumor immunity by stimulating dendritic cell activation, T cell priming, and immune-mediated tumor clearance. Recent studies have revealed that PRMT3 can suppress innate immune signaling by regulating mitochondrial stress pathways. Specifically, PRMT3 has been shown to methylate the mitochondrial chaperone protein heat shock protein 60 (HSP60). This post-translational modification promotes the oligomerization and stabilization of HSP60, which plays an important role in maintaining mitochondrial homeostasis (95).

Through the methylation of HSP60, PRMT3 prevents mitochondrial stress-induced release of mitochondrial DNA (mtDNA) into the cytosol. Because cytosolic DNA is a major trigger of cGAS activation, preventing mtDNA release suppresses the activation of the cGAS–STING signaling pathway (96–98). As a result, the production of interferons and pro-inflammatory cytokines is reduced, leading to diminished innate immune activation. Suppression of the cGAS–STING pathway can significantly impair anti-tumor immune responses (99). By preventing the activation of innate immune signaling, PRMT3 limits immune cell recruitment and reduces the ability of the immune system to recognize and eliminate tumor cells. This mechanism represents an additional strategy by which PRMT3 contributes to tumor immune escape.

Interestingly, inhibition of PRMT3 has been shown to restore cGAS–STING signaling and enhance anti-tumor immunity. Loss of PRMT3 activity leads to reduced HSP60 methylation, increased mitochondrial stress, and the release of mtDNA into the cytosol, which subsequently activates cGAS–STING signaling. This activation promotes immune cell infiltration and enhances anti-tumor immune responses, highlighting the potential therapeutic value of targeting PRMT3 in cancer immunotherapy.

### 6.4 Immunometabolic crosstalk

The mechanisms described above illustrate how PRMT3 integrates metabolic regulation and immune suppression to promote tumor progression. Tumor metabolism and immune responses are tightly interconnected processes within the tumor microenvironment, and alterations in metabolic pathways can profoundly influence immune cell function.

PRMT3-mediated metabolic reprogramming contributes to immune suppression through several mechanisms. Activation of glycolysis leads to increased lactate production, which inhibits the activity of cytotoxic immune cells and promotes an immunosuppressive tumor microenvironment. At the same time, suppression of innate immune sensing pathways such as cGAS–STING prevents the activation of immune responses that would otherwise target tumor cells (100, 101). Through these coordinated mechanisms, PRMT3 establishes a metabolic and immunological landscape that favors tumor survival and immune evasion. By simultaneously regulating metabolic enzymes, immune checkpoint molecules, and innate immune signaling pathways, PRMT3 functions as a central mediator of immunometabolic crosstalk in cancer.

**Figure 5:**
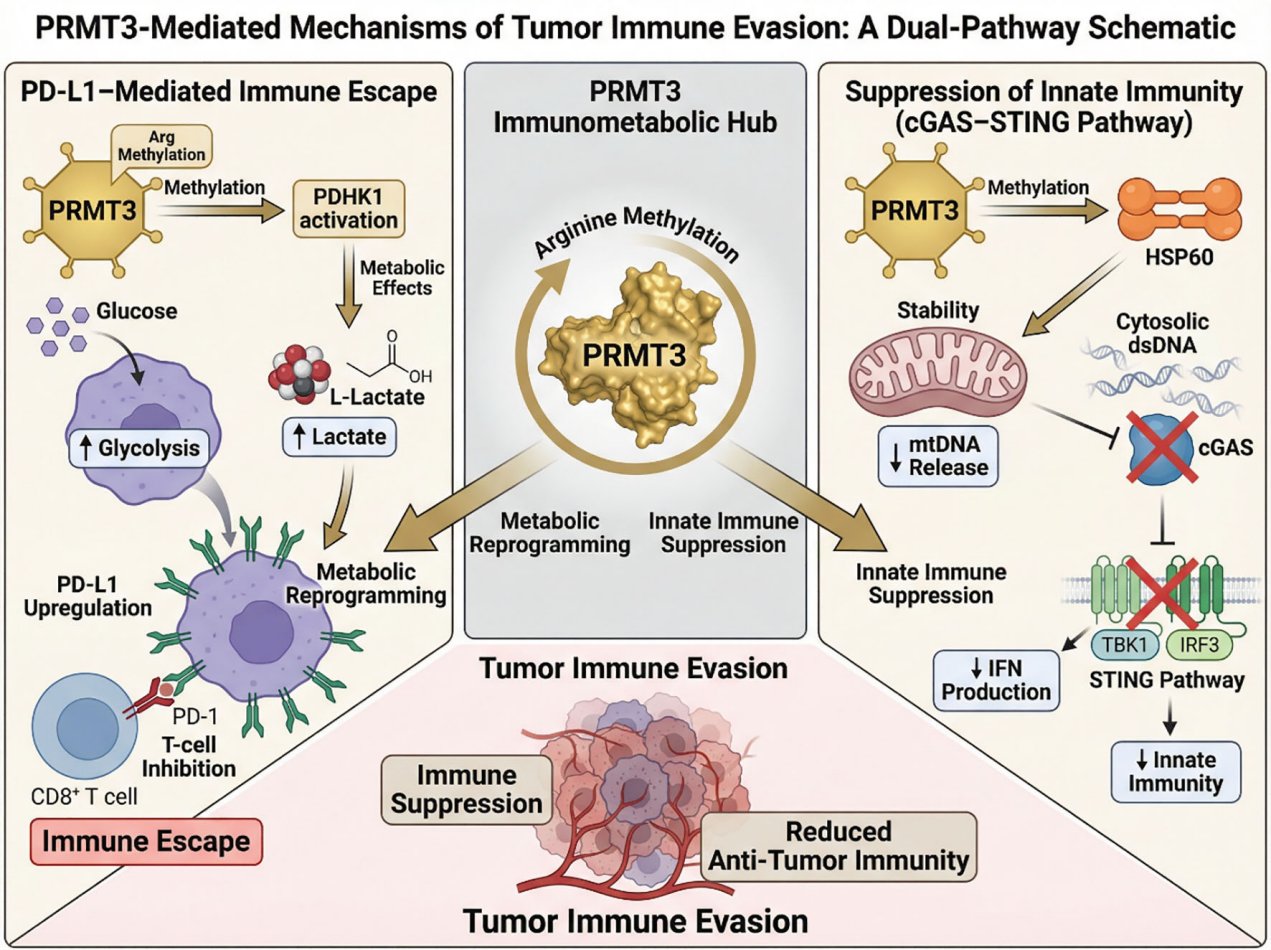
PRMT3 promotes tumor immune evasion through coordinated metabolic and innate immune regulatory pathways. PRMT3-driven activation of PDHK1 enhances glycolysis and induces PD-L1 expression, suppressing CD8^+^ T-cell activity. In addition, PRMT3-mediated methylation of HSP60 inhibits mitochondrial DNA release and prevents activation of the cGAS–STING pathway. These mechanisms collectively create an immunosuppressive tumor microenvironment that facilitates tumor immune escape.

These findings highlight the importance of PRMT3 as a molecular bridge connecting tumor metabolism and immune regulation. Targeting PRMT3-mediated signaling pathways may therefore represent a promising strategy to restore anti-tumor immunity and enhance the efficacy of immunotherapy (**Figure 5**).

## 7 PRMT3 in therapy resistance

Resistance to anticancer therapy remains one of the major challenges in oncology and is a primary cause of treatment failure and tumor recurrence. Cancer cells can acquire resistance to chemotherapy, radiotherapy, and targeted therapies through a variety of mechanisms, including altered drug metabolism, enhanced DNA damage repair, metabolic adaptation, and immune suppression. Increasing evidence suggests that post-translational modifications play a crucial role in these adaptive processes. Among these regulatory mechanisms, arginine methylation mediated by PRMT3 has recently emerged as an important contributor to therapeutic resistance in cancer. Recent studies have demonstrated that PRMT3 promotes resistance to anticancer therapies through multiple pathways. These include stabilization of oncogenic transcripts through RNA-binding proteins, activation of immunosuppressive metabolic pathways, and enhancement of drug efflux mechanisms. Through these mechanisms, PRMT3 enables cancer cells to adapt to therapeutic stress and maintain survival under treatment conditions.

### 7.1 Chemoresistance

Chemotherapy remains a cornerstone of cancer treatment, but the development of chemoresistance frequently limits its long-term effectiveness (102, 103). Tumor cells can develop resistance to chemotherapeutic agents through multiple mechanisms, including altered drug uptake, activation of survival signaling pathways, and stabilization of oncogenic transcripts. Recent studies have revealed that PRMT3 contributes to chemoresistance by regulating RNA-binding proteins that control mRNA stability. One important example is insulin-like growth factor 2 mRNA-binding protein 1 (IGF2BP1), a well-characterized RNA-binding protein that stabilizes oncogenic mRNAs and promotes tumor progression (104, 105). IGF2BP1 functions as a reader of m6A-modified transcripts and plays a critical role in maintaining the stability and translation of multiple cancer-associated mRNAs.

PRMT3-mediated arginine methylation has been shown to enhance the stability and activity of IGF2BP1. Methylation of IGF2BP1 increases its ability to bind target transcripts and protect them from degradation. As a result, oncogenic mRNAs involved in cell survival, proliferation, and stress adaptation remain stabilized within tumor cells. This mechanism has been directly linked to resistance to oxaliplatin chemotherapy in hepatocellular carcinoma. Oxaliplatin is commonly used in the treatment of liver cancer, but resistance to this drug frequently develops during therapy. PRMT3-mediated stabilization of IGF2BP1 enhances the expression of survival-related transcripts, allowing tumor cells to tolerate chemotherapeutic stress. By protecting oncogenic mRNAs from degradation, PRMT3 effectively rewires post-transcriptional gene regulation to support chemoresistant phenotypes. These findings highlight a novel mechanism by which PRMT3 regulates chemotherapy resistance through the modulation of RNA regulatory networks. By controlling the stability of oncogenic transcripts, PRMT3 enables tumor cells to maintain key survival pathways even in the presence of cytotoxic drugs.

### 7.2 Radioresistance

Radiotherapy is widely used for the treatment of solid tumors and functions primarily by inducing DNA damage and promoting immune-mediated tumor destruction (106–108). However, many tumors develop resistance to radiation therapy through adaptive cellular responses that enhance survival and suppress immune activation.

Recent studies have identified PRMT3 as an important regulator of radioresistance through its control of immunometabolic pathways. One key mechanism involves the activation of indoleamine 2,3-dioxygenase 1 (IDO1), a metabolic enzyme that catalyzes the conversion of tryptophan into kynurenine. Activation of the IDO1–kynurenine pathway is known to create an immunosuppressive tumor microenvironment that inhibits T cell activity and promotes tumor immune escape. PRMT3 has been shown to drive the expression and activity of IDO1 in cancer cells, thereby enhancing tryptophan metabolism and increasing kynurenine production. Elevated kynurenine levels suppress cytotoxic T cell responses and promote the expansion of immunosuppressive immune cell populations. This immunosuppressive environment reduces the effectiveness of radiation-induced immune responses, allowing tumor cells to survive despite DNA damage induced by radiotherapy. In addition to suppressing anti-tumor immunity, activation of the IDO1 pathway may also promote tumor cell survival through metabolic adaptation. The kynurenine pathway produces several metabolites that support tumor growth and regulate oxidative stress responses. Through these mechanisms, PRMT3-mediated activation of IDO1 contributes to both immune suppression and metabolic resilience in cancer cells.

Together, these findings demonstrate that PRMT3 plays a key role in promoting resistance to radiotherapy by modulating immunometabolic pathways. By activating the IDO1–kynurenine axis, PRMT3 suppresses anti-tumor immune responses and enhances tumor cell survival under radiation-induced stress.

### 7.3 Drug efflux and multidrug resistance

Another major mechanism of chemotherapy resistance involves the active efflux of drugs from cancer cells. Multidrug resistance (MDR) transporters, particularly members of the ATP-binding cassette (ABC) transporter family, can export chemotherapeutic agents out of tumor cells and reduce intracellular drug concentrations (109–111). Among these transporters, ABCG2 is widely recognized as an important mediator of multidrug resistance in several cancer types. Recent studies have demonstrated that PRMT3 can regulate drug efflux pathways through the methylation of RNA-binding proteins. In pancreatic cancer, PRMT3-mediated arginine methylation of heterogeneous nuclear ribonucleoprotein A1 (hnRNPA1) enhances its ability to regulate gene expression. hnRNPA1 is an RNA-binding protein involved in RNA splicing, mRNA transport, and translational regulation. Methylation of hnRNPA1 by PRMT3 increases its stability and transcriptional regulatory activity, leading to the upregulation of ABCG2 expression. Elevated ABCG2 levels enhance the ability of tumor cells to export chemotherapeutic drugs, thereby reducing intracellular drug accumulation and promoting chemoresistance. This PRMT3–hnRNPA1–ABCG2 regulatory axis illustrates how arginine methylation can influence multidrug resistance mechanisms at the post-transcriptional level (112–114). By enhancing the expression of drug transporters, PRMT3 enables tumor cells to actively eliminate cytotoxic drugs and maintain survival during chemotherapy.

### 7.4 PRMT3 as a central regulator of therapy resistance

Taken together, these findings indicate that PRMT3 contributes to therapy resistance through multiple complementary mechanisms. By stabilizing oncogenic transcripts through IGF2BP1, activating immunosuppressive metabolic pathways via IDO1, and promoting drug efflux through hnRNPA1 and ABCG2, PRMT3 enables cancer cells to adapt to diverse therapeutic pressures (115–119). Importantly, these resistance mechanisms are interconnected with the broader functions of PRMT3 in RNA regulation, metabolic reprogramming, and immune suppression. This integrative role suggests that PRMT3 functions as a central regulator of therapeutic resistance in cancer. Targeting PRMT3-mediated signaling pathways may therefore represent a promising strategy for overcoming therapy resistance and improving the efficacy of anticancer treatments.

## 8 Therapeutic targeting of PRMT3

Given the emerging role of PRMT3 in tumor progression, metabolic reprogramming, immune evasion, and therapeutic resistance, targeting PRMT3 has attracted increasing interest as a potential anticancer strategy. As a type I protein arginine methyltransferase responsible for catalyzing asymmetric dimethylation of arginine residues, PRMT3 regulates multiple oncogenic signaling pathways through post-translational modification of key proteins. Dysregulation of PRMT3 activity has been implicated in several malignancies, suggesting that pharmacological inhibition of PRMT3 could disrupt tumor-promoting signaling networks. Although the therapeutic targeting of PRMT3 remains in the early stages compared with other PRMT family members such as PRMT1 or PRMT5, recent advances in drug discovery have begun to explore both small-molecule inhibitors and targeted protein degradation strategies. These emerging approaches provide promising opportunities for developing PRMT3-based anticancer therapies. Current therapeutic strategies targeting PRMT3 are summarized in **Table 3**.

**Table 3:** Therapeutic strategies targeting PRMT3 in cancer.

Strategy	Representative agent/modality	Mechanism	Key pharmacologic features/evidence	Therapeutic implication	Reference
PRMT3 enzymatic inhibition	SGC707	Selective allosteric inhibition of PRMT3 catalytic activity	First well-characterized PRMT3 chemical probe; IC50 = 31 ± 2 nM, KD = 53 ± 2 nM; high selectivity over 31 other methyltransferases and >250 non-epigenetic targets; cell-active and suitable for *in vivo* chemical biology studies	Suppresses PRMT3-dependent oncogenic signaling and provides a pharmacologic basis for PRMT3-targeted therapy	Kaniskan et al., 2015 (22)
Optimized PRMT3 allosteric inhibitors	SGC707-derived analogs/optimized allosteric compounds	Allosteric inhibition of PRMT3	Structure–activity relationship studies identified additional potent compounds targeting the PRMT3 allosteric pocket, with biochemical IC50 values in the ~10–36 nM range	Supports the druggability of PRMT3 and provides leads for future medicinal chemistry optimization	Kaniskan et al., 2018 (120)
PRMT3 targeted degradation	PRMT3 degrader 11	PROTAC-mediated selective degradation of PRMT3	First-in-class PRMT3-targeted degrader reported in acute leukemia; induces selective PRMT3 degradation and showed stronger anti-leukemia growth inhibition than SGC707 in preclinical settings	Suggests that targeted PRMT3 elimination may achieve deeper pathway suppression than catalytic inhibition alone	Zou et al., *Adv Sci*, 2024 (121)
Combination with chemotherapy	SGC707 + oxaliplatin	PRMT3 inhibition reduces PRMT3-mediated chemoresistance	In HCC, pharmacologic inhibition of PRMT3 counteracted PRMT3-driven phenotypes and restored sensitivity to oxaliplatin	May improve chemotherapy efficacy in PRMT3-high or oxaliplatin-resistant tumors	Shi et al., *Nat Commun*, 2023 (64)
Combination with immunotherapy	PRMT3 inhibition + immune checkpoint blockade	Relieves PRMT3-associated immune suppression and may restore anti-tumor immunity	Mechanistically supported by studies linking PRMT3 to PD-L1 regulation and immune escape, but direct drug-combination efficacy data remain limited	Potential strategy to enhance immunotherapy response in PRMT3-high tumors	Preclinical rationale; emerging evidence
Combination with radiotherapy	PRMT3 targeting + radiotherapy	May overcome PRMT3-associated radioresistance and immune suppression	Rationale supported by PRMT3-linked survival and stress-adaptation pathways, but pharmacologic validation remains limited	Potential radiosensitization strategy requiring further validation	Early preclinical rationale

### 8.1 PRMT3 inhibitors

Small-molecule inhibitors targeting methyltransferases have attracted considerable interest as potential anticancer therapeutics (122, 123). Although several inhibitors against other PRMT family members, particularly PRMT1 and PRMT5, have advanced into clinical evaluation, the development of selective PRMT3 inhibitors remains comparatively less mature (34, 120, 124). Importantly, current PRMT3 inhibitor development has been driven not only by catalytic-site considerations, but more prominently by the identification of a druggable allosteric pocket, which has enabled the discovery of highly selective chemical probes.

The best-characterized PRMT3 inhibitor is SGC707, a potent, selective, and cell-active allosteric inhibitor. Biochemical and structural studies showed that SGC707 inhibits PRMT3 with an IC50 of 31 ± 2 nM and a KD of 53 ± 2 nM, while displaying excellent selectivity over 31 other methyltransferases and more than 250 non-epigenetic targets (22). These findings established PRMT3 as a pharmacologically tractable target and provided a valuable tool for mechanistic and preclinical studies. Subsequent medicinal chemistry efforts further identified optimized allosteric analogs with biochemical IC50 values in the ~10–36 nM range, supporting the feasibility of targeting the PRMT3 allosteric site for selective inhibitor development. Emerging preclinical evidence suggests that pharmacological inhibition of PRMT3 may suppress tumor-promoting phenotypes in selected cancer contexts (51, 64, 65). In hepatocellular carcinoma, for example, SGC707 has been shown to counteract PRMT3-driven oncogenic effects and restore sensitivity to oxaliplatin, supporting the therapeutic relevance of PRMT3 inhibition in chemotherapy-resistant tumors (14). In addition, mechanistic studies suggest that PRMT3 inhibition may attenuate glycolytic reprogramming and partially relieve immunosuppressive signaling, although these effects still require broader validation across tumor types.

Despite these advances, several challenges remain. Most currently available PRMT3 inhibitors are still chemical probes or early preclinical compounds, and comprehensive data on pharmacokinetics, *in vivo* efficacy, long-term tolerability, and tumor-type selectivity remain limited. Therefore, although PRMT3 inhibition represents a promising therapeutic strategy, further structural, pharmacological, and translational studies are needed before PRMT3 inhibitors can be considered clinically mature anticancer agents.

### 8.2 PRMT3 degraders

In addition to enzymatic inhibitors, targeted protein degradation has emerged as a powerful strategy for eliminating disease-associated proteins. Unlike conventional inhibitors that block catalytic activity, targeted degraders promote the complete removal of the target protein from cells, potentially resulting in more durable therapeutic effects. Recent advances in targeted protein degradation technologies, such as proteolysis-targeting chimeras (PROTACs), have enabled the development of degraders targeting epigenetic regulators and signaling proteins. These molecules function by recruiting the target protein to an E3 ubiquitin ligase, leading to ubiquitination and subsequent degradation by the proteasome. A recent study reported the discovery of a PRMT3 degrader with potential therapeutic applications in acute leukemia (121). This degrader promotes the selective degradation of PRMT3 protein in leukemia cells, resulting in the suppression of oncogenic signaling pathways and inhibition of tumor cell proliferation. Compared with traditional enzyme inhibitors, targeted degradation of PRMT3 may provide additional advantages by eliminating both catalytic and non-catalytic functions of the protein. Targeted degradation strategies are particularly attractive for proteins like PRMT3 that participate in multiple signaling pathways. By removing PRMT3 entirely, degraders may simultaneously disrupt metabolic regulation, RNA regulatory networks, and immune suppression pathways that contribute to tumor progression. Although PRMT3 degraders are still in early stages of development, these studies demonstrate the feasibility of targeting PRMT3 through protein degradation technologies. Continued research in this area may lead to the development of more effective and selective therapeutic agents.

### 8.3 Combination therapeutic strategies

Given the multifaceted roles of PRMT3 in cancer biology, combining PRMT3-targeted therapies with other treatment modalities may enhance therapeutic efficacy. PRMT3 participates in metabolic regulation, immune suppression, and therapy resistance pathways, suggesting that its inhibition could sensitize tumors to conventional therapies and immunotherapies.

One promising approach involves combining PRMT3 inhibition with immune checkpoint blockade. Because PRMT3 can promote PD-L1 expression and suppress innate immune signaling pathways such as cGAS–STING, inhibition of PRMT3 may restore anti-tumor immune responses and enhance the efficacy of immune checkpoint inhibitors (125, 126). By reducing immunosuppressive signaling within the tumor microenvironment, PRMT3 inhibitors may improve T cell activation and tumor immune clearance. Another potential strategy is the combination of PRMT3 inhibitors with chemotherapy. PRMT3-mediated stabilization of oncogenic transcripts through RNA-binding proteins such as IGF2BP1 contributes to chemotherapy resistance. Inhibiting PRMT3 could destabilize these transcripts and sensitize tumor cells to cytotoxic drugs, thereby improving chemotherapy responses. Similarly, PRMT3 inhibition may enhance the effectiveness of radiotherapy. As described earlier, PRMT3-driven activation of the IDO1–kynurenine pathway contributes to radioresistance by suppressing anti-tumor immunity. Blocking PRMT3 activity could reduce immunosuppressive signaling and restore immune-mediated tumor killing following radiation treatment.

Taken together, these findings suggest that PRMT3-targeted therapies may be most effective when used in combination with existing treatment strategies. By disrupting multiple oncogenic pathways simultaneously, combination therapies targeting PRMT3 may provide a more comprehensive approach to cancer treatment.

## 9 Challenges and future perspectives

Although substantial progress has been made in defining the oncogenic functions of PRMT3 in cancer, several critical questions remain insufficiently resolved. Beyond simply expanding the list of PRMT3 substrates, future studies should address more specific mechanistic and translational issues. For example, it remains unclear whether PRMT3 exhibits different substrate-recognition preferences in normal versus tumor cells, whether systemic PRMT3 inhibition may cause clinically significant on-target toxicities in immune, hematopoietic, or other normal tissues, and how PRMT3-targeted therapeutics can be delivered more selectively to tumors. Addressing these questions will be essential for improving both the biological understanding and translational feasibility of PRMT3-targeted strategies.

### 9.1 Incomplete characterization of the PRMT3 substrate network

One of the major challenges in PRMT3 research is the incomplete characterization of its substrate repertoire. Arginine methylation can influence numerous cellular processes by modifying proteins involved in transcription, RNA metabolism, signal transduction, and metabolic regulation. Although several PRMT3 substrates have been identified—including IGF2BP1, METTL14, HIF1A, hnRNPA1, and HSP60—the full spectrum of PRMT3 targets remains largely unknown (127–129). Comprehensive identification of PRMT3 substrates is essential for understanding how this enzyme regulates complex oncogenic signaling networks. Advances in proteomic technologies, such as methylarginine-specific mass spectrometry and quantitative methyl-proteomics, provide powerful tools for identifying new PRMT3 substrates on a global scale. Integrating these approaches with functional genomic screening methods, including CRISPR-based screening platforms, may help uncover novel regulatory pathways controlled by PRMT3. A more complete mapping of the PRMT3 substrate network will not only deepen our understanding of its biological functions but also reveal new molecular mechanisms underlying tumor progression and therapeutic resistance.

### 9.2 PTM crosstalk as a mechanistic basis for PRMT3-mediated protein stabilization

In the current literature, PRMT3-mediated arginine methylation is frequently described as promoting the “stabilization” of oncogenic proteins such as HIF1A and c-MYC. However, protein stabilization is unlikely to represent a simple descriptive outcome and instead may reflect post-translational modification (PTM) crosstalk. In principle, arginine methylation may prolong protein half-life by altering local charge, protein conformation, or the accessibility of nearby residues, thereby affecting polyubiquitination and ubiquitin-dependent degradation. For HIF1A, available evidence indicates that PRMT3-mediated methylation is associated with decreased polyubiquitination without altering hydroxylation, suggesting that methylation interferes with ubiquitin-dependent turnover rather than the upstream hydroxylation step itself (42, 53). For c-MYC, PRMT3 has likewise been reported to reduce polyubiquitination and increase protein stability, although the exact molecular basis remains insufficiently defined (41). These observations support the concept that PRMT3 exerts its stabilizing effects, at least in part, through PTM crosstalk with the ubiquitin-proteasome system. Future studies should further determine whether PRMT3-mediated arginine methylation affects E3 ligase recognition, steric accessibility, or cooperativity with other modifications such as phosphorylation and acetylation.

### 9.3 Context-dependent functions of PRMT3 across cancer types

Another important challenge is the context-dependent role of PRMT3 in different tumor types. Although PRMT3 is generally considered to function as an oncogenic regulator, its specific mechanisms of action may vary depending on the cellular and metabolic context of individual cancers. For example, PRMT3 promotes glycolytic metabolism through HIF1A stabilization in glioblastoma and colorectal cancer, while in hepatocellular carcinoma it drives glycolytic activation through the PDHK1 pathway. In non-small cell lung cancer, PRMT3 has been shown to regulate tryptophan metabolism through the IDO1–kynurenine pathway, leading to immunosuppression and radioresistance. These observations suggest that PRMT3 can regulate distinct signaling pathways depending on the tumor microenvironment and metabolic demands of different cancers. In addition, the functional impact of PRMT3 may be influenced by interactions with other epigenetic regulators, transcription factors, and metabolic enzymes. Understanding how PRMT3 integrates into these broader regulatory networks will be essential for identifying tumor-specific vulnerabilities that can be therapeutically exploited. Future studies combining multi-omics approaches—including transcriptomics, proteomics, metabolomics, and epigenomics—may provide deeper insights into the context-specific roles of PRMT3 in cancer biology.

### 9.4 Limited development of selective PRMT3 inhibitors

Despite growing interest in PRMT3 as a therapeutic target, the development of selective PRMT3 inhibitors remains limited. Compared with other PRMT family members such as PRMT1 and PRMT5, which have already been explored in clinical trials, PRMT3-targeted drug discovery is still in its early stages (34, 35). One of the main challenges lies in the structural similarity shared among PRMT family members, particularly within their catalytic domains. This similarity makes it difficult to design inhibitors that selectively target PRMT3 without affecting other PRMT enzymes. Off-target inhibition of related PRMTs could lead to unintended biological effects and limit the clinical applicability of these compounds. Recent advances in structure-guided drug design and computational modeling may help overcome these challenges by enabling the development of more selective inhibitors targeting unique structural features of PRMT3. In addition, targeted protein degradation strategies, such as PROTAC-based degraders, may offer an alternative approach for eliminating PRMT3 activity in cancer cells. Further research focusing on structural biology and drug discovery will be critical for advancing PRMT3-targeted therapies toward clinical translation.

### 9.5 PRMT3 as a potential target for cancer immunotherapy

Recent discoveries linking PRMT3 to immune regulation have raised the possibility that this enzyme may represent a novel target for cancer immunotherapy. PRMT3 influences multiple immunological pathways, including PD-L1 expression, immunometabolic regulation through the IDO1–kynurenine axis, and suppression of innate immune signaling via the cGAS–STING pathway. These mechanisms collectively contribute to the establishment of an immunosuppressive tumor microenvironment (130). Targeting PRMT3 may therefore enhance anti-tumor immunity by simultaneously modulating several immune regulatory pathways. For instance, inhibition of PRMT3 could reduce PD-L1 expression, restore cytotoxic T cell activity, and activate innate immune signaling pathways that promote immune cell recruitment. Such effects could potentially enhance the efficacy of immune checkpoint inhibitors and other immunotherapeutic approaches. However, the immunological consequences of PRMT3 inhibition remain incompletely understood. Because PRMT enzymes also regulate normal immune cell function, it will be important to carefully evaluate the effects of PRMT3-targeted therapies on immune homeostasis (131–134). Future studies using immunocompetent tumor models and clinical datasets will be necessary to determine whether PRMT3 inhibition can effectively enhance anti-tumor immunity without causing adverse immune-related effects.

### 9.6 Key questions for future investigation

Several key questions should be prioritized in future PRMT3 research. First, does PRMT3 recognize different substrates in normal and malignant cells, and what molecular determinants govern this context dependence? Second, what are the major on-target toxicities of systemic PRMT3 inhibition, particularly in immune, hematopoietic, and metabolically active tissues? Third, can tumor-targeted delivery systems improve the selectivity and safety of PRMT3 inhibitors or degraders? Finally, how can patient subsets most likely to benefit from PRMT3-targeted therapy be identified on the basis of substrate dependence, immunometabolic phenotype, or combination-treatment vulnerability? Addressing these questions will help move the field beyond descriptive substrate mapping and toward mechanism-based and clinically actionable translation.

## 10 Conclusion

Accumulating evidence over the past decade suggests that protein arginine methyltransferase 3 (PRMT3) is increasingly implicated in cancer biology. Originally characterized as a methyltransferase involved in ribosomal protein modification, PRMT3 is now recognized as an enzyme with functions extending beyond ribosome-associated processes. Through arginine methylation of selected regulatory proteins, PRMT3 has been linked to multiple cellular pathways relevant to tumor progression and therapeutic response. Recent studies indicate that PRMT3 may participate in several major regulatory networks in cancer. At the epigenetic and post-transcriptional levels, PRMT3 has been reported to regulate RNA-binding proteins and epitranscriptomic regulators, thereby influencing RNA stability and gene expression programs associated with tumor growth. In addition, PRMT3 appears to contribute to metabolic reprogramming by promoting glycolytic activity and modulating amino acid metabolism, which may help tumor cells adapt to metabolic stress within the tumor microenvironment. PRMT3 has also been associated with tumor immune evasion through effects on immune checkpoint pathways and innate immune signaling mechanisms. Importantly, current evidence for these functions is derived from different cancer types, experimental systems, and study settings, and they have not yet been systematically validated as a unified regulatory network within the same biological context. Thus, PRMT3 may be better viewed as an emerging and context-dependent coordinator, rather than a definitively established central hub linking metabolism, immune suppression, and therapeutic adaptation. Furthermore, PRMT3 has been implicated in several forms of therapy resistance, including chemoresistance, radioresistance, and multidrug resistance. These observations suggest that PRMT3 may contribute not only to tumor progression but also to reduced sensitivity to anticancer therapies in specific settings. Accordingly, targeting PRMT3 could represent a promising strategy for overcoming treatment resistance and improving therapeutic outcomes, although further mechanistic and translational studies are needed. Continued efforts to define PRMT3-associated signaling pathways and to develop selective inhibitors or degraders may provide new opportunities for therapeutic intervention. Overall, PRMT3 represents a promising candidate therapeutic target and a potential biomarker for precision oncology.
